# Novel Meta-Analysis-Derived Type 2 Diabetes Risk Loci Do Not Determine Prediabetic Phenotypes

**DOI:** 10.1371/journal.pone.0003019

**Published:** 2008-08-20

**Authors:** Harald Staiger, Fausto Machicao, Konstantinos Kantartzis, Silke A. Schäfer, Kerstin Kirchhoff, Martina Guthoff, Günther Silbernagel, Norbert Stefan, Andreas Fritsche, Hans-Ulrich Häring

**Affiliations:** Department of Internal Medicine, Division of Endocrinology, Diabetology, Angiology, Nephrology, and Clinical Chemistry, University Hospital Tübingen, Tübingen, Germany; National Institute of Child Health and Human Development/National Institutes of Health, United States of America

## Abstract

**Background:**

Genome-wide association (GWA) studies identified a series of novel type 2 diabetes risk loci. Most of them were subsequently demonstrated to affect insulin secretion of pancreatic β-cells. Very recently, a meta-analysis of GWA data revealed nine additional risk loci with still undefined roles in the pathogenesis of type 2 diabetes. Using our thoroughly phenotyped cohort of subjects at an increased risk for type 2 diabetes, we assessed the association of the nine latest genetic variants with the predominant prediabetes traits, i.e., obesity, impaired insulin secretion, and insulin resistance.

**Methodology/Principal Findings:**

One thousand five hundred and seventy-eight metabolically characterized non-diabetic German subjects were genotyped for the reported candidate single nucleotide polymorphisms (SNPs) *JAZF1* rs864745, *CDC123/CAMK1D* rs12779790, *TSPAN8/LGR5* rs7961581, *THADA* rs7578597, *ADAMTS9* rs4607103, *NOTCH2* rs10923931, *DCD* rs1153188, *VEGFA* rs9472138, and *BCL11A* rs10490072. Insulin sensitivity was derived from fasting glucose and insulin concentrations, oral glucose tolerance test (OGTT), and hyperinsulinemic-euglycemic clamp. Insulin secretion was estimated from OGTT data. After appropriate adjustment for confounding variables and Bonferroni correction for multiple comparisons (corrected α-level: p = 0.0014), none of the SNPs was reliably associated with adiposity, insulin sensitivity, or insulin secretion (all p≥0.0117, dominant inheritance model). The risk alleles of *ADAMTS9* SNP rs4607103 and *VEGFA* SNP rs9472138 tended to associate with more than one measure of insulin sensitivity and insulin secretion, respectively, but did not reach formal statistical significance. The study was sufficiently powered (1-β = 0.8) to detect effect sizes of 0.19≤d≤0.25 (α = 0.0014) and 0.13≤d≤0.16 (α = 0.05).

**Conclusions/Significance:**

In contrast to the first series of GWA-derived type 2 diabetes candidate SNPs, we could not detect reliable associations of the novel risk loci with prediabetic phenotypes. Possible weak effects of *ADAMTS9* SNP rs4607103 and *VEGFA* SNP rs9472138 on insulin sensitivity and insulin secretion, respectively, await further confirmation by larger studies.

## Introduction

Type 2 diabetes mellitus results from an interaction between environmental factors, such as high-caloric nutrition and reduced physical activity, and a predisposing polygenic background. More explicitly, common variation within several genes is thought to confer enhanced susceptibility towards the aforementioned environmental challenges [1]. During the pathogenesis of type 2 diabetes, peripheral tissues, such as liver, skeletal muscle, and adipose tissue, develop insulin resistance which provokes compensatory increments in pancreatic insulin secretion. When insulin resistance reaches extents no longer compensated by the β-cell, insulin secretion declines and hyperglycemia emerges [2]. Thus, genetic variation in type 2 diabetes risk genes is supposed to affect insulin sensitivity and/or β-cell function.

Last year, genome-wide association (GWA) studies based on several thousands of cases and controls not only confirmed the importance of earlier type 2 diabetes candidate genes, such as *PPARG*, *KCNJ11*, and *TCF7L2*, but also identified single nucleotide polymorphisms (SNPs) within five novel risk loci, i.e., *SLC30A8*, *HHEX*, *CDKAL1*, *IGF2BP2*, and *CDKN2A/B* [3]–[6]. The association of the novel loci with type 2 diabetes was subsequently reproduced in several other cohorts and ethnicities [7]–[14]. Furthermore, analysis of cohorts phenotyped with state-of-the-art methods for measurement of insulin sensitivity and insulin secretion recently revealed that the novel genetic variants affect insulin secretion, but not insulin sensitivity [10;15–21].

In a very recent meta-analysis of GWA data, nine additional risk loci were identified with equal or weaker association with type 2 diabetes (odds ratios: 1.05–1.15) as compared to the first series of novel risk loci (odds ratios: 1.12–1.37) [22]. The role of the corresponding genes, i.e., *JAZF1*, *CDC123/CAMK1D*, *TSPAN8/LGR5*, *THADA*, *ADAMTS9*, *NOTCH2*, *DCD*, *VEGFA*, and *BCL11A*, in the pathogenesis of prediabetes phenotypes was not yet assessed and is not established in the literature. Therefore, it was the aim of the present study to test the association of the nine most recent candidate SNPs with obesity, insulin resistance, and β-cell dysfunction in a thoroughly metabolically characterized population at an increased risk for type 2 diabetes from Southern Germany.

## Methods

### Subjects

One thousand seven hundred and twenty subjects were recruited from the ongoing Tübingen Family Study for type 2 diabetes (TÜF) which currently includes ∼2000 individuals. The publicly announced call for TÜF primarily addressed non-diabetic individuals from Southern Germany at an increased risk for type 2 diabetes (family history of type 2 diabetes, diagnosis of impaired fasting glycemia). More than 99.5% of the TÜF participants are of European ancestry. Selection of the study cohort was based on availability of DNA samples and C-peptide measurements. From the 1720 subjects, 45 were excluded due to incomplete data sets and 97 due to newly diagnosed type 2 diabetes. These exclusions resulted in a non-diabetic cohort of 1578 subjects (1139 with normal glucose tolerance, 164 with impaired fasting glycemia, 152 with impaired glucose tolerance, and 123 with impaired fasting glycemia and impaired glucose tolerance). 68% of these subjects had a recorded family history of type 2 diabetes, i.e., at least one 2^nd^-degree relative with type 2 diabetes. All participants underwent the standard procedures of the protocol including medical history and physical examination, assessment of smoking status, alcohol consumption habits and activity, routine blood tests, and oral glucose tolerance test (OGTT). A subgroup of 513 subjects agreed to undergo a hyperinsulinemic-euglycemic clamp. The participants were not taking any medication known to affect glucose tolerance or insulin secretion. Informed written consent to the study was obtained from the participants, and the local ethics committee (Ethik-Kommission der Medizinischen Fakultät der Universität Tübingen) approved the study protocol.

### Genotyping of the study population

For genotyping, DNA was isolated from whole blood using a commercial DNA isolation kit (NucleoSpin, Macherey & Nagel, Düren, Germany). SNPs were genotyped using the TaqMan assay (Applied Biosystems, Foster City, CA, USA). The TaqMan genotyping reaction was amplified on a GeneAmp PCR system 7000 (50°C for 2 min, 95°C for 10 min, followed by 40 cycles of 95°C for 15s and 60°C for 1 min), and fluorescence was detected on an ABI Prism sequence detector (Applied Biosystems, Foster City, CA, USA). The assay was validated by bidirectional sequencing in 50 subjects, and both methods gave 100% identical results. The overall genotyping success rate was 99.5% (rs864745: 99.7%, rs12779790: 97.8%, rs7961581: 99.4%, rs7578597: 99.9%, rs4607103: 99.2%, rs10923931: 100%, rs1153188: 99.7%, rs9472138: 99.7%, rs10490072: 99.9%), and rescreening of 3.2% of subjects resulted in 100% identical results.

### Determination of adiposity

Percentage of body fat was measured by bioelectrical impedance (BIA-101, RJL systems, Detroit, MI, USA). Body mass index (BMI) was calculated as weight divided by squared height. Waist circumference was measured in the upright position at the midpoint between the lateral iliac crest and the lowest rib.

### OGTT

After a 10h overnight fast, all subjects underwent a 75g OGTT and venous blood samples were obtained at 0, 30, 60, 90, and 120min for determination of plasma glucose, insulin, and C-peptide.

### Hyperinsulinemic-euglycemic clamp

After an overnight fast and a 60min baseline period, 513 subjects received a priming dose of insulin followed by an infusion (40mU/m^2^) of short-acting human insulin for 120 min. A variable infusion of 20% glucose was started to maintain the fasting plasma glucose concentration. Blood samples for the measurement of plasma glucose were obtained at 5 min intervals throughout the clamp. Plasma insulin levels were measured at baseline and in the steady state of the clamp.

### Determination of blood parameters

Plasma glucose was determined using a bedside glucose analyzer (glucose oxidase method, Yellow Springs Instruments, Yellow Springs, CO, USA). Plasma insulin and C-peptide concentrations were measured by commercial chemiluminescence assays for ADVIA Centaur (Siemens Medical Solutions, Fernwald, Germany) according to the manufacturer's instructions. The inter-assay coefficients of variation were <5% (insulin assay) and <6% (C-peptide assay).

### Calculations

The area under the curve (AUC) of plasma glucose levels during the OGTT was calculated according to the trapezoid method as: 0.5·[0.5·c(glucose)_0_+c(glucose)_30_+c(glucose)_60_+c(glucose)_90_+0.5·c(glucose)_120_]. The AUC of plasma C-peptide levels during the OGTT was calculated analogously. Insulin secretion in the OGTT was assessed by calculating the ratio of the AUC of C-peptide divided through the AUC of glucose (AUC C-pep/AUC glc). First-phase insulin secretion was estimated from plasma insulin and glucose concentrations during the OGTT using the formerly described equation [23]: 1,283+1.829·c(insulin)_30_-138.7·c(glucose)_30_+3.772·c(insulin)_0_. Homeostasis model assessment of insulin resistance (HOMA-IR) was calculated as [c(glucose)_0_·c(insulin)_0_]/22.5. Insulin sensitivity from OGTT was estimated as proposed by Matsuda and DeFronzo [24]: 10,000/[c(glucose)_0_·c(insulin)_0_·c(glucose)_mean_·c(insulin)_mean_]^½^. Clamp-derived insulin sensitivity was calculated as glucose infusion rate necessary to maintain euglycemia during the last 40min (steady state) of the clamp divided by the steady-state insulin concentration.

### Statistical analyses

Hardy-Weinberg equilibrium was tested using χ^2^ test. Prior to statistical analysis, all continuous data were log-transformed in order to approximate normal distribution. To adjust for confounding variables, multivariate linear regression models were applied, and the trait of interest (e.g., BMI, insulin sensitivity index, or insulin secretion index) was chosen as dependent variable. Multivariate linear regression analysis was performed using the least-squares method. Based on testing nine non-linked SNPs and four independent parameters (i.e., age, measures of adiposity, measures of insulin secretion, and measures of insulin action), we performed 36 independent statistical tests. Therefore, a p-value<0.0014 was considered statistically significant according to Bonferroni correction for multiple comparisons. For these analyses, the statistical software package JMP 4.0 (SAS Institute, Cary, NC, USA) was used. In the dominant inheritance model using one-tailed t-test, our study was sufficiently powered (1-β = 0.8, α = 0.0014) to detect effect sizes (d) of 0.19≤d≤0.25 depending on the minor allele frequency (MAF) of the SNP tested. At the nominal α-level of 0.05, the study was sufficiently powered to detect effect sizes of 0.13≤d≤0.16. In the subgroup of clamped subjects, the study was sufficiently powered to detect effect sizes of 0.34≤d≤0.46 (1-β = 0.8, α = 0.0014) or of 0.22≤d≤0.30 (1-β = 0.8, α = 0.05). Power calculations were performed using G*power 3.0 software available at http://www.psycho.uni-duesseldorf.de/aap/projects/gpower/.

## Results

We genotyped 1578 non-diabetic subjects (clinical characteristics given in Table 1) for the following type 2 diabetes candidate SNPs: the intronic SNP rs864745 in the *JAZF1* gene (chr. 7), SNP rs12779790 located in the genomic region between the *CDC123* and *CAMK1D* genes (chr. 10), SNP rs7961581 located between *TSPAN8* and *LGR5* (chr. 12), SNP rs7578597 in exon 24 of the *THADA* gene (chr. 2) resulting in the missense mutation T1187A, the intronic SNP rs4607103 in the *ADAMTS9* gene (chr. 3), the intronic SNP rs10923931 in the *NOTCH2* gene (chr. 1; in near-complete linkage disequilibrium with SNP rs2641348 in the *ADAM30* gene [22]), SNP rs1153188 nearest (5′-flanking) to the *DCD* gene (chr. 12), SNP rs9472138 nearest (3′-flanking) to the *VEGFA* gene (chr. 6), and SNP rs10490072 nearest (3′-flanking) to the *BCL11A* gene (chr. 2). All SNPs were in Hardy-Weinberg equilibrium (all p>0.1) and displayed MAFs similar to those recently reported [22] (Tables 2, 3, 4).

**Table 1:** Clinical characteristics of the study population (N = 1578: 1139 NGT, 164 IFG, 152 IGT, 123 IFG+IGT).

	women (N = 1044)		men (N = 534)	
	mean±SE	range	mean±SE	range
Age (y)	39±0	14–80	40±1	17–91
BMI (kg/m^2^)	29.2±0.3	16.3–76.9	28.3±0.3	17.6–69.7
Body fat (%)	35±0	9–67	23±0	7–62
Waist circumference (cm)	91±1	56–178	99±1	52–183
Fasting glucose (mM)	5.07±0.02	3.00–6.94	5.17±0.02	3.50–7.00
Glucose 120min OGTT (mM)	6.36±0.05	2.44–11.06	6.09±0.08	2.50–11.06
Fasting insulin (pM)	65.6±1.7	10.0–614.0	60.1±2.2	8.0–373.0
Insulin 120min OGTT (pM)	454±14	43–6422	392±20	10–4475

IFG–impaired fasting glycemia; IGT–impaired glucose tolerance; NGT–normal glucose tolerance.

**Table 2:** Associations of *JAZF1* SNP rs864745, *CDC123/CAMK1D* SNP rs12779790, and *TSPAN8/LGR5* SNP rs7961581 with anthropometrics, insulin sensitivity, and insulin secretion (N = 1578).

SNP (MAF)	*JAZF1* rs864745 (0.48)					*CDC123/CAMK1D* rs12779790 (0.18)					*TSPAN8/LGR5* rs7961581 (0.31)				
Genotype	AA	AG	GG	p_1_	p_2_	AA	AG	GG	p_1_	p_2_	TT	TC	CC	p_1_	p_2_
N	440	750	379	-	-	1031	466	45	-	-	750	669	147	-	-
Age (y)	39±13	40±14	39±13	0.4	0.2	40±13	39±14	40±13	0.6	0.5	39±13	40±14	38±12	0.8	0.9
BMI (kg/m^2^)	28.7±8.1	29.0±8.1	28.8±8.5	0.8	0.6	28.9±8.3	28.4±7.7	28.6±9.7	0.5	0.3	28.7±7.8	29.0±8.7	28.9±8.0	0.9	0.7
Body fat (%)	31±11	31±11	30±11	0.3	0.8	31±11	31±11	30±11	0.3	0.8	31±11	31±11	32±10	0.6	0.5
Waist circum-ference (cm)	93±16	94±18	94±18	0.6	0.8	94±17	94±17	91±18	0.6	1.0	94±17	94±18	94±16	0.9	0.9
Fasting glucose (mM)	5.06±0.57	5.14±0.56	5.09±0.50	0.2	0.1	5.10±0.55	5.10±0.55	5.14±0.41	0.5	0.4	5.11±0.54	5.10±0.55	5.10±0.59	0.7	0.5
Glucose 120min OGTT (mM)	6.23±1.68	6.31±1.67	6.20±1.61	0.8	1.0	6.24±1.63	6.28±1.68	6.39±1.88	0.6	0.3	6.26±1.61	6.25±1.70	6.35±1.71	0.7	0.8
ISI, OGTT (U)	16.7±11.3	15.7±10.4	17.2±10.9	0.0260	0.6	16.2±10.6	16.4±10.8	20.8±13.8	0.06	0.9	16.0±10.7	16.9±11.0	15.5±10.0	0.0219	0.06
ISI, clamp (U)*	0.088±0.059	0.081±0.047	0.091±0.065	1.0	0.9	0.082±0.051	0.090±0.061	0.109±0.077	0.5	0.9	0.082±0.053	0.090±0.058	0.079±0.048	0.2	0.5
HOMA-IR (U)	2.51±2.24	2.56±2.35	2.28±1.94	0.0260	0.5	2.45±2.16	2.52±2.36	2.20±2.41	0.1	0.8	2.53±2.30	2.36±2.10	2.64±2.34	0.0042	0.0138
1^st^-phase insulin secretion (nM)	1.29±0.81	1.28±0.85	1.24±0.87	0.6	0.4	1.29±0.86	1.25±0.83	1.05±0.74	0.4	0.3	1.29±0.86	1.24±0.82	1.30±0.82	0.9	0.7
C-peptide 30min OGTT (nM)	2.05±0.85	2.07±0.91	2.02±0.91	1.0	0.8	2.06±0.90	2.07±0.87	1.67±0.68	0.0398	0.7	2.06±0.90	2.04±0.88	2.07±0.91	0.5	0.6
AUC C-pep/AUC glc (·10^−9^)	323±107	323±111	310±103	0.5	0.3	320±108	325±105	272±75	0.0423	0.5	323±108	316±105	324±112	0.9	0.7

Data represent means±SD. For statistical analysis, data were log-transformed. Age was adjusted for gender. BMI, body fat, and waist circumference were adjusted for gender and age. Plasma glucose levels and indices of insulin sensitivity were adjusted for gender, age, and BMI. Indices of insulin secretion were adjusted for gender, age, BMI, and ISI (OGTT). p_1_–p-value, additive model; p_2_–p-value, dominant model. AUC–area under the curve; HOMA-IR–homeostasis model assessment of insulin resistance; ISI–insulin sensitivity index; MAF–minor allele frequency; SNP–single nucleotide polymorphism.

* subgroup (N = 513).

**Table 3:** Associations of *THADA* SNP rs7578597, *ADAMTS9* SNP rs4607103, and *NOTCH2* SNP rs10923931# with anthropometrics, insulin sensitivity, and insulin secretion (N = 1578).

SNP (MAF)	*THADA* rs7578597 (0.11)					*ADAMTS9* rs4607103 (0.27)					*NOTCH2* rs10923931 (0.10)				
Genotype	TT	TC	CC	p_1_	p_2_	CC	CT	TT	p_1_	p_2_	GG	GT	TT	p_1_	p_2_
N	1246	305	22	-	-	834	619	111	-	-	1293	260	22	-	-
Age (y)	40±13	39±13	40±14	0.4	0.2	40±13	39±13	39±14	0.2	0.08	40±13	39±13	39±14	0.8	0.5
BMI (kg/m^2^)	28.6±8.0	29.8±9.0	30.4±7.4	0.0370	0.0117	28.5±7.6	29.4±8.7	28.9±9.3	0.08	0.0377	29.0±8.2	28.4±8.2	28.3±8.6	0.5	0.2
Body fat (%)	31±11	32±11	34±9	0.1	0.06	31±11	31±11	30±11	0.2	0.7	31±11	30±10	32±12	0.3	0.1
Waist circum-ference (cm)	93±17	96±19	97±16	0.05	0.0177	94±17	95±18	91±17	0.2	0.5	94±17	93±19	95±20	0.5	0.4
Fasting glucose (mM)	5.10±0.55	5.12±0.56	5.11±0.53	1.0	0.9	5.12±0.55	5.08±0.55	5.09±0.51	0.2	0.1	5.10±0.55	5.11±0.56	5.14±0.54	0.6	0.3
Glucose 120min OGTT (mM)	6.30±1.67	6.10±1.61	6.34±1.47	0.05	0.0173	6.34±1.69	6.19±1.63	6.13±1.49	0.2	0.07	6.29±1.68	6.21±1.51	5.86±1.66	0.4	0.9
ISI, OGTT (U)	16.4±10.8	16.0±10.3	16.2±11.7	0.6	0.3	16.3±10.8	16.1±10.5	17.4±11.7	0.2	0.07	16.2±10.8	16.7±10.7	16.6±10.5	1.0	0.9
ISI, clamp (U)*	0.086±0.058	0.082±0.044	0.081±0.037	0.9	0.8	0.083±0.050	0.088±0.061	0.093±0.060	0.3	0.2	0.085±0.053	0.090±0.064	0.096±0.048	0.9	0.7
HOMA-IR (U)	2.40±2.05	2.75±2.81	2.71±2.37	0.8	0.8	2.52±2.31	2.46±2.14	2.31±2.17	0.0351	0.0125	2.48±2.19	2.46±2.43	2.64±2.04	0.6	0.7
1^st^-phase insulin secretion (nM)	1.26±0.80	1.33±1.01	1.32±0.89	0.9	0.7	1.24±0.81	1.32±0.89	1.22±0.80	0.7	0.5	1.28±0.86	1.22±0.78	1.32±0.66	0.4	0.9
C-peptide 30min OGTT (nM)	2.04±0.87	2.09±0.95	2.16±1.16	1.0	0.8	2.03±0.87	2.09±0.91	1.98±0.93	0.4	0.4	2.06±0.91	2.02±0.86	2.03±0.59	0.8	0.9
AUC C-pep/AUC glc (·10^−9^)	319±108	324±108	326±103	0.9	0.6	320±105	321±109	317±120	0.9	0.6	321±110	316±98	325±80	0.8	0.8

Data represent means±SD. For statistical analysis, data were log-transformed. Age was adjusted for gender. BMI, body fat, and waist circumference were adjusted for gender and age. Plasma glucose levels and indices of insulin sensitivity were adjusted for gender, age, and BMI. Indices of insulin secretion were adjusted for gender, age, BMI, and ISI (OGTT). p_1_–p-value, additive model; p_2_–p-value, dominant model. AUC–area under the curve; HOMA-IR–homeostasis model assessment of insulin resistance; ISI–insulin sensitivity index; MAF–minor allele frequency; SNP–single nucleotide polymorphism.

* subgroup (N = 513).

# in linkage with *ADAM30* SNP rs2641348.

**Table 4:** Associations of *DCD* SNP rs1153188, *VEGFA* SNP rs9472138, and *BCL11A* SNP rs10490072 with anthropometrics, insulin sensitivity, and insulin secretion (N = 1578).

SNP (MAF)	*DCD* rs1153188 (0.25)					*VEGFA* rs9472138 (0.30)					*BCL11A* rs10490072 (0.26)				
Genotype	TT	TA	AA	p_1_	p_2_	CC	CT	TT	p_1_	p_2_	TT	TC	CC	p_1_	p_2_
N	879	580	110	-	-	772	650	148	-	-	835	644	95	-	-
Age (y)	39±13	40±14	40±14	0.5	0.3	39±13	39±14	41±13	0.4	0.7	38±13	41±14	40±13	0.0066	0.0017
BMI (kg/m^2^)	28.9±8.0	29.0±8.7	28.0±7.2	0.5	0.6	29.0±8.3	28.6±8.0	28.8±8.9	0.6	0.3	28.9±8.3	28.9±8.2	28.1±7.1	0.7	0.6
Body fat (%)	31±11	31±11	30±11	0.0260	0.0119	31±11	31±11	31±11	0.6	0.3	31±11	31±11	30±10	0.5	0.5
Waist circum-ference (cm)	94±17	94±18	93±18	0.7	0.4	94±18	93±16	95±19	0.4	0.3	94±18	94±17	92±15	0.8	0.8
Fasting glucose (mM)	5.10±0.56	5.13±0.53	5.06±0.58	0.3	0.5	5.10±0.54	5.11±0.54	5.11±0.60	0.6	0.6	5.10±0.54	5.11±0.57	5.07±0.50	0.4	0.2
Glucose 120min OGTT (mM)	6.27±1.70	6.29±1.61	6.11±1.58	0.6	0.9	6.23±1.65	6.34±1.67	6.18±1.62	0.1	0.2	6.25±1.61	6.29±1.73	6.21±1.52	0.8	0.5
ISI, OGTT (U)	16.2±10.7	16.6±10.9	16.1±10.1	0.4	0.4	16.4±11.2	16.2±10.3	16.6±10.5	0.9	0.8	16.5±10.8	16.2±10.6	15.6±11.2	0.6	0.4
ISI, clamp (U)*	0.081±0.050	0.091±0.061	0.090±0.053	0.1	0.1	0.089±0.056	0.082±0.054	0.086±0.050	0.2	0.08	0.085±0.056	0.087±0.054	0.078±0.054	0.5	0.7
HOMA-IR (U)	2.51±2.20	2.45±2.33	2.30±1.81	0.6	0.4	2.56±2.34	2.38±2.04	2.33±2.04	0.7	0.5	2.48±2.32	2.48±2.16	2.36±1.73	0.6	0.4
1^st^-phase insulin secretion (nM)	1.29±0.88	1.23±0.76	1.29±0.93	0.2	0.5	1.30±0.89	1.23±0.76	1.27±0.92	0.4	0.2	1.28±0.87	1.26±0.81	1.27±0.82	0.7	0.9
C-peptide 30min OGTT (nM)	2.07±0.91	2.01±0.88	2.14±0.87	0.06	0.8	2.11±0.92	1.99±0.83	2.04±1.00	0.07	0.0269	2.05±0.89	2.06±0.90	2.10±0.83	0.5	0.5
AUC C-pep/AUC glc (·10^−9^)	323±110	313±104	336±108	0.0288	0.6	327±112	312±100	319±111	0.0497	0.0268	317±106	322±109	332±107	0.2	0.1

Data represent means±SD. For statistical analysis, data were log-transformed. Age was adjusted for gender. BMI, body fat, and waist circumference were adjusted for gender and age. Plasma glucose levels and indices of insulin sensitivity were adjusted for gender, age, and BMI. Indices of insulin secretion were adjusted for gender, age, BMI, and ISI (OGTT). p_1_–p-value, additive model; p_2_–p-value, dominant model. AUC–area under the curve; HOMA-IR–homeostasis model assessment of insulin resistance; ISI–insulin sensitivity index; MAF–minor allele frequency; SNP–single nucleotide polymorphism.

* subgroup (N = 513).

After appropriate adjustment for confounding variables and Bonferroni correction for multiple comparisons (corrected α-level: p = 0.0014), none of the SNPs was reliably associated with age, adiposity, plasma glucose concentrations, insulin sensitivity, or insulin secretion either in the additive (all p≥0.0042) or in the dominant inheritance model (all p≥0.0017), as presented in Tables 2, 3, 4. Since our cohort included twice as many women as men and adjusting for gender might not completely account for this difference, we performed analyses in women and men separately. However, these analyses did not provide more reliable associations than the analysis of the total study population (Tables S1, S2, S3, S4, S5, S6).

By analysing each SNP for trends of association (arbitrary α-level: p = 0.07, dominant inheritance model) with more than one measure of adiposity, insulin sensitivity, or insulin secretion, respectively, the following trends were observed: the *TSPAN8/LGR5* SNP rs7961581 tended to associate with OGTT-derived insulin sensitivity and HOMA-IR, the *THADA* SNP rs7578597 with BMI, body fat content, and waist circumference, the *ADAMTS9* SNP rs4607103 with OGTT-derived insulin sensitivity and HOMA-IR, and the *VEGFA* SNP rs9472138 with C-peptide levels at 30 min of OGTT and AUC C-pep/AUC glc. After determination of the risk alleles for these associations, only the risk allele of the *ADAMTS9* SNP rs4607103 and the risk allele of the *VEGFA* SNP rs9472138 were identical with the recently reported risk alleles for type 2 diabetes [22].

## Discussion

In our thoroughly phenotyped cohort, we could recently demonstrate that several of the type 2 diabetes candidate SNPs identified in the course of the first round of GWA analysis were significantly associated with β-cell dysfunction and/or impaired proinsulin-to-insulin conversion [15], [16]. In the present study, we assessed the association of the nine latest candidate SNPs identified by recent meta-analysis of GWA data [22] with prediabetic traits. As compared to the first series, these latest SNPs displayed only very weak association with type 2 diabetes (odds ratios: 1.05–1.15) [22] and, thus, might also include false-positives. Taking this suggestion and the large number of statistical tests performed into account, we rigorously applied Bonferroni correction for multiple comparisons in order to minimize the number of statistical type 1 errors. By analysing the data in this way, we could not detect any reliable association of the candidate SNPs with the prediabetes traits obesity, insulin resistance, or impaired insulin secretion.

By analysing each SNP for trends of association (arbitrary α-level: p = 0.07, dominant inheritance model), the risk alleles of the *ADAMTS9* SNP rs4607103 and the *VEGFA* SNP rs9472138 tended to associate with more than one measure of insulin sensitivity and insulin secretion, respectively. Thus, one could speculate that genetic variation within *ADAMTS9* and *VEGFA* may exert weak effects on these traits. To corroborate these findings, further studies in larger and comparably well-phenotyped cohorts are needed which allow the reliable detection of effect sizes smaller than d = 0.19. However, the clinical relevance of such small effects remains to be determined.

A very recently published study investigated associations of the type 2 diabetes risk alleles in *JAZF1*, *CDC123/CAMK1D*, *TSPAN8/LGR5*, *THADA*, *ADAMTS9*, and *NOTCH2* with obesity, insulin sensitivity, and insulin secretion in 4516 Danes [25]. This study confirms our negative findings for a role of these SNPs in adiposity and insulin sensitivity as well as our negative results for a role of the *THADA*, *ADAMTS9*, and *NOTCH2* SNPs in insulin secretion. However, using OGTT-based estimates of insulin secretion derived from plasma insulin and glucose levels, these authors demonstrated associations of the *JAZF1*, *CDC123/CAMK1D*, and *TSPAN8/LGR5* SNPs with insulin secretion. Only the association of *CDC123/CAMK1D* SNP rs12779790 with the insulinogenic index resisted Bonferroni correction for multiple comparisons. Even though we feel that C-peptide measurements, as performed in our study, are more reliable in estimating insulin release than insulin data, which are biased by insulin resistance and insulin clearance, our study is of limited statistical power and our negative findings (which could reflect statistical type 2 errors) are therefore insufficient to reject a possible role of genetic variation in *JAZF1*, *CDC123/CAMK1D*, or *TSPAN8/LGR5* in β-cell dysfunction. Thus, larger studies with C-peptide measurements or studies using more sophisticated methods for the measurement of insulin secretion, such as the intravenous glucose tolerance test or the hyperglycemic clamp, are needed to ultimately address this issue. Alternative measures of insulin secretion in addition to the OGTT-derived measures reported in the present and the former study [25] are particularly important in order to capture all of the various aspects of insulin secretion capacity.

In conclusion, none of the tested candidate SNPs displayed significant association with crucial prediabetes phenotypes. Since it is highly plausible that type 2 diabetes candidate SNPs affect adiposity, insulin sensitivity, or insulin secretion, our negative findings could point to the possibility that these SNPs' associations with type 2 diabetes in part reflect statistical type 1 errors. Possible weak effects of *ADAMTS9* SNP rs4607103 and *VEGFA* SNP rs9472138 on insulin sensitivity and insulin secretion, respectively, cannot be excluded and await further confirmation by larger studies.

## Supporting Information

Table S1 Supplementary Table 1
(0.06 MB DOC)

Table S2 Supplementary Table 2
(0.06 MB DOC)

Table S3 Supplementary Table 3
(0.06 MB DOC)

Table S4 Supplementary Table 4
(0.06 MB DOC)

Table S5 Supplementary Table 5
(0.06 MB DOC)

Table S6 Supplementary Table 6
(0.06 MB DOC)
