# Supplementary material for: Novel Meta-Analysis-Derived Type 2 Diabetes Risk Loci Do Not Determine Prediabetic Phenotypes
Source: PLoS One. 2008 Aug 20;3(8):e3019. doi: 10.1371/journal.pone.0003019 (PMC2500187; doi:10.1371/journal.pone.0003019)
Supplement: Table S2 — Supplementary Table 2 (0.06 MB DOC) [file pone.0003019.s002.doc]

**Table S2.** Associations of *THADA* SNP rs7578597, *ADAMTS9* SNP rs4607103, and *NOTCH2* SNP rs10923931^#^ with anthropometrics, insulin sensitivity, and insulin secretion in women only (N=1044).

| SNP | *THADA* rs7578597 | | | | | *ADAMTS9* rs4607103 | | | | | *NOTCH2* rs10923931 | | | | |
| --- | --- | --- | --- | --- | --- | --- | --- | --- | --- | --- | --- | --- | --- | --- | --- |
| Genotype | TT | TC | CC | p_1_ | p_2_ | CC | CT | TT | p_1_ | p_2_ | GG | GT | TT | p_1_ | p_2_ |
| N | 823 | 200 | 17 | - | - | 558 | 402 | 74 | - | - | 852 | 172 | 17 | - | - |
| Age (y) | 39 ±13 | 39 ±13 | 40 ±12 | 0.9 | 0.8 | 40 ±13 | 38 ±13 | 39 ±14 | 0.2 | 0.06 | 39 ±13 | 38 ±13 | 39 ±15 | 0.6 | 0.3 |
| BMI (kg/m²) | 28.9 ±8.3 | 30.1 ±9.0 | 29.8 ±7.2 | 0.2 | 0.07 | 28.7 ±7.9 | 29.7 ±9.0 | 29.3 ±9.8 | 0.2 | 0.1 | 29.4 ±8.6 | 28.1 ±7.8 | 28.8 ±9.6 | 0.2 | 0.07 |
| Body fat (%) | 35 ±10 | 36 ±10 | 36 ±8 | 0.4 | 0.2 | 35 ±10 | 35 ±10 | 34 ±10 | 0.7 | 0.9 | 35 ±10 | 33 ±9 | 35 ±12 | 0.2 | 0.08 |
| Waist circum-ference (cm) | 91 ±16 | 93 ±18 | 93 ±15 | 0.1 | 0.0437 | 91 ±16 | 92 ±17 | 88 ±15 | 0.2 | 1.0 | 92 ±17 | 89 ±16 | 95 ±22 | 0.2 | 0.2 |
| Fasting glucose (mM) | 5.07 ±0.54 | 5.07 ±0.55 | 5.05 ±0.29 | 0.7 | 0.4 | 5.10 ±0.54 | 5.02 ±0.53 | 5.09 ±0.57 | 0.0316 | 0.0232 | 5.07 ±0.53 | 5.05 ±0.55 | 5.16 ±0.54 | 0.6 | 0.6 |
| Glucose 120min OGTT (mM) | 6.39 ±1.62 | 6.24 ±1.57 | 6.20 ±1.30 | 0.2 | 0.06 | 6.46 ±1.61 | 6.25 ±1.58 | 6.24 ±1.63 | 0.05 | 0.0151 | 6.39 ±1.63 | 6.22 ±1.44 | 6.09 ±1.65 | 0.7 | 0.6 |
| ISI, OGTT (U) | 15.9 ±10.2 | 15.7 ±10.0 | 16.0 ±11.7 | 0.5 | 0.2 | 15.6 ±9.8 | 16.0 ±10.6 | 17.0 ±10.7 | 0.05 | 0.0159 | 15.5 ±10.2 | 17.0 ±10.1 | 16.3 ±10.8 | 0.4 | 0.2 |
| HOMA-IR (U) | 2.47 ±2.09 | 2.72 ±2.69 | 2.28 ±1.41 | 0.8 | 0.8 | 2.57 ±2.31 | 2.52 ±2.15 | 2.25 ±1.85 | 0.0323 | 0.0115 | 2.57 ±2.23 | 2.26 ±2.12 | 2.78 ±2.27 | 0.5 | 0.4 |
| 1^st^-phase insulin secretion (nM) | 1.29 ±0.80 | 1.30 ±0.91 | 1.22 ±0.59 | 0.8 | 0.5 | 1.27 ±0.81 | 1.34 ±0.84 | 1.23 ±0.76 | 0.3 | 0.1 | 1.31 ±0.84 | 1.17 ±0.69 | 1.33 ±0.74 | 0.6 | 0.5 |
| C-peptide 30min OGTT (nM) | 2.01 ±0.84 | 2.05 ±0.90 | 2.08 ±0.72 | 0.9 | 0.8 | 2.01 ±0.85 | 2.05 ±0.86 | 1.91 ±0.86 | 0.3 | 0.4 | 2.03 ±0.87 | 1.95 ±0.80 | 2.02 ±0.62 | 0.9 | 0.8 |
| AUC C-pep/AUC glc (·10^-9^) | 323 ±107 | 326 ±104 | 325 ±79 | 0.7 | 0.5 | 323 ±104 | 327 ±106 | 312 ±114 | 0.4 | 0.7 | 325 ±108 | 316 ±92 | 325 ±84 | 1.0 | 0.9 |

Data represent means ±SD. For statistical analysis, data were log-transformed. BMI, body fat, and waist circumference were adjusted for age. Plasma glucose levels and indices of insulin sensitivity were adjusted for age and BMI. Indices of insulin secretion were adjusted for age, BMI, and ISI (OGTT). p_1_ – p-value, additive model; p_2_ – p-value, dominant model. AUC – area under the curve; HOMA-IR – homeostasis model assessment of insulin resistance; ISI – insulin sensitivity index; SNP – single nucleotide polymorphism. ^#^in linkage with *ADAM30* SNP rs2641348.
