# Supplementary material for: Novel Meta-Analysis-Derived Type 2 Diabetes Risk Loci Do Not Determine Prediabetic Phenotypes
Source: PLoS One. 2008 Aug 20;3(8):e3019. doi: 10.1371/journal.pone.0003019 (PMC2500187; doi:10.1371/journal.pone.0003019)
Supplement: Table S4 — Supplementary Table 4 (0.06 MB DOC) [file pone.0003019.s004.doc]

**Table S4.** Associations of *JAZF1* SNP rs864745, *CDC123/CAMK1D* SNP rs12779790, and *TSPAN8/LGR5* SNP rs7961581 with anthropometrics, insulin sensitivity, and insulin secretion in men only (N=534).

| SNP | *JAZF1* rs864745 | | | | | *CDC123/CAMK1D* rs12779790 | | | | | *TSPAN8/LGR5* rs7961581 | | | | |
| --- | --- | --- | --- | --- | --- | --- | --- | --- | --- | --- | --- | --- | --- | --- | --- |
| Genotype | AA | AG | GG | p_1_ | p_2_ | AA | AG | GG | p_1_ | p_2_ | TT | TC | CC | p_1_ | p_2_ |
| N | 144 | 256 | 133 | - | - | 358 | 152 | 10 | - | - | 248 | 238 | 47 | - | - |
| Age (y) | 39 ±14 | 42 ±14 | 39 ±13 | 0.0435 | 0.07 | 41 ±14 | 39 ±14 | 42 ±15 | 0.3 | 0.2 | 41 ±14 | 40 ±14 | 41 ±14 | 0.6 | 0.4 |
| BMI (kg/m²) | 27.7 ±7.1 | 28.4 ±7.5 | 28.7 ±8.5 | 0.6 | 0.5 | 28.4 ±7.7 | 27.8 ±7.2 | 26.7 ±4.6 | 0.7 | 0.5 | 28.0 ±7.2 | 28.3 ±7.7 | 29.4 ±9.2 | 0.6 | 0.4 |
| Body fat (%) | 23 ±8 | 23 ±8 | 23 ±7 | 1.0 | 1.0 | 23 ±8 | 23 ±8 | 21 ±6 | 0.7 | 0.6 | 22 ±7 | 24 ±8 | 24 ±8 | 0.1 | 0.0454 |
| Waist circum-ference (cm) | 97 ±17 | 99 ±17 | 100 ±20 | 0.5 | 0.4 | 99 ±18 | 98 ±18 | 97 ±14 | 0.8 | 0.9 | 99 ±18 | 99 ±18 | 100 ±16 | 0.9 | 0.8 |
| Fasting glucose (mM) | 5.12 ±0.61 | 5.22 ±0.59 | 5.15 ±0.47 | 0.6 | 0.5 | 5.16 ±0.57 | 5.19 ±0.57 | 5.15 ±0.33 | 0.3 | 0.1 | 5.16 ±0.52 | 5.16 ±0.59 | 5.29 ±0.66 | 0.6 | 0.8 |
| Glucose 120min OGTT (mM) | 6.05 ±1.88 | 6.11 ±1.74 | 6.10 ±1.63 | 0.8 | 0.8 | 6.03 ±1.69 | 6.13 ±1.68 | 6.21 ±1.71 | 0.5 | 0.2 | 6.04 ±1.66 | 6.11 ±1.82 | 6.20 ±1.80 | 0.9 | 0.7 |
| ISI, OGTT (U) | 17.7 ±12.7 | 16.7 ±11.2 | 17.5 ±11.9 | 0.5 | 0.3 | 17.3 ±11.9 | 16.8 ±11.2 | 25.2 ±17.9 | 0.07 | 0.3 | 17.5 ±12.3 | 17.3 ±11.5 | 15.2 ±9.8 | 0.8 | 0.8 |
| HOMA-IR (U) | 2.41 ±2.27 | 2.41 ±2.37 | 2.32 ±2.03 | 0.3 | 0.2 | 2.36 ±2.16 | 2.53 ±2.55 | 1.62 ±1.26 | 0.0415 | 0.2 | 2.37 ±2.21 | 2.31 ±2.14 | 2.86 ±2.95 | 0.4 | 0.5 |
| 1^st^-phase insulin secretion (nM) | 1.21 ±0.78 | 1.22 ±0.84 | 1.28 ±1.05 | 0.8 | 0.7 | 1.28 ±0.93 | 1.20 ±0.81 | 0.81 ±0.40 | 0.2 | 0.08 | 1.23 ±0.81 | 1.24 ±0.95 | 1.29 ±0.92 | 0.3 | 0.1 |
| C-peptide 30min OGTT (nM) | 2.09 ±0.91 | 2.11 ±0.96 | 2.17 ±1.04 | 0.8 | 0.6 | 2.16 ±1.00 | 2.09 ±0.86 | 1.46 ±0.49 | 0.09 | 0.1 | 2.06 ±0.86 | 2.16 ±1.01 | 2.27 ±1.24 | 0.7 | 0.4 |
| AUC C-pep/AUC glc (·10^-9^) | 313 ±102 | 317 ±115 | 306 ±110 | 0.6 | 0.8 | 316 ±112 | 315 ±100 | 237 ±49 | 0.3 | 0.5 | 311 ±100 | 315 ±116 | 327 ±134 | 1.0 | 0.9 |

Data represent means ±SD. For statistical analysis, data were log-transformed. BMI, body fat, and waist circumference were adjusted for age. Plasma glucose levels and indices of insulin sensitivity were adjusted for age and BMI. Indices of insulin secretion were adjusted for age, BMI, and ISI (OGTT). p_1_ – p-value, additive model; p_2_ – p-value, dominant model. AUC – area under the curve; HOMA-IR – homeostasis model assessment of insulin resistance; ISI – insulin sensitivity index; SNP – single nucleotide polymorphism.
